# Influence of Bed Depth on the Development of Tropical Ornamental Plants in Subsurface Flow Treatment Wetlands for Municipal Wastewater Treatment: A Pilot-Scale Case

**DOI:** 10.3390/plants13141958

**Published:** 2024-07-17

**Authors:** Graciela Nani, Mayerlin Sandoval-Herazo, Georgina Martínez-Reséndiz, Oscar Marín-Peña, Florentina Zurita, Luis Carlos Sandoval Herazo

**Affiliations:** 1 Wetlands and Environmental Sustainability Laboratory, Division of Graduate Studies and Research, Tecnológico Nacional de Meéxico/Instituto Tecnológico Superior de Misantla, Km 1.8, Carretera a Loma del Cojolite, Misantla 93821, Veracruz, Mexico; 2 Postdoctoral Program, CONAHCYT (Consejo Nacional de Ciencia Humanidades y Tecnología), Tecnológico Nacional de México Campus Misantla, Misantla 93821, Veracruz, Mexico; 3 Environmental Quality Research Center, Centro Universitario de la Ciénega, University of Guadalajara, Av. Universidad 1115, Ocotlán 47820, Jalisco, Mexico

**Keywords:** *Alpinia purpurata*, *Heliconia latispatha*, *Strelitzia reginae*, ornamental plants, saturation level, subsurface flow treatment wetlands

## Abstract

The aim of this 2-year study was to evaluate the influence of bed depth (40 and 60 cm) on the development of tropical ornamental species (*Alpinia purpurata, Heliconia latispatha* and *Strelitzia reginae*) and on the removal of different contaminants such as chemical oxygen demand (COD), nitrate (N-NO_3_), ammonium (N-NH_4_), total nitrogen (TN), total phosphorus (TP), total suspended solids (TSS), total coliforms (TCs) and fecal coliforms (FCs), in horizontal subsurface flow constructed wetlands (HSSF-CWs) for municipal wastewater treatment. The results showed that the depth of 60 cm favored the removal of COD, with removal efficiencies of 94% for the three plant species. The depth of 40 cm was most effective for the removal of N-NH_4_ (80–90%). Regarding the removal of TN, the removals were similar for the different plants and depths (72–86%). The systems only achieved up to 60% removal of TCs and FCs. The depth of the CWs substrate and its saturation level influenced the development of ornamental vegetation, particularly flower production. For *Heliconia latispatha*, a bed depth level of 60 cm was more suitable, while for *Alpinia purpurata* 40 cm was better, and for *Strelitzia reginae* in both cases there was no flower production. The impact of bed depth on contaminant removal depends on the specific type of contaminant.

## 1. Introduction

Constructed wetlands (CWs) are engineered systems designed to take advantage of natural wetland processes in the transformation and removal of pollutants, but under controlled conditions. Thus, the construction of facilities with precise combinations of substrates, vegetation types and flow patterns is possible [1]. CWs are green, affordable and reliable technologies for the treatment of various types of wastewaters. Due to limited economic resources in developing countries and the high costs of construction, operation and maintenance of sanitation infrastructure, CWs represent a low-cost option for rural and urban wastewater treatment, which could help reduce the current health gap between developed and developing countries [2,3]. Additionally, the use of ornamental vegetations that produce flowers in CWs can give an aesthetically pleasing appearance to the systems, allowing their incorporation into landscapes in urban and rural environments. They can also be used as a business strategy, since they can be considered as cultivation areas for ornamental vegetations of commercial interest [4,5].

The efficiency of CWs and contaminant removal mechanisms varies depending on different factors, such as the particular design, the type of wastewater to be treated, environmental conditions and operational management [6]. In horizontal subsurface flow constructed wetlands (HSSF-CWs), wastewater moves slowly below the surface of a filter bed filled with porous material and emergent plants. This design creates aerobic, anoxic and anaerobic zones as the wastewater interacts with the filter material.

The aerobic zones are confined to narrow areas near the roots and rhizomes of plants that release oxygen into the substrate, while anoxic/anaerobic conditions prevail in the rest of the system due to the saturated hydraulic regime. These conditions enable efficient removal of organic matter, achieving chemical oxygen demand (COD) removal rates between 89% and 98.46%, and total suspended solids (TSS) rates between 78% and 94.8%. Additionally, they promote denitrification for total nitrogen (TN) removal, reaching removal rates of up to 90%, depending on the wastewater characteristics and operating conditions [7].

On the other hand, the bed depth in HSSF-CWs determines the internal conditions that will prevail. In general, shallower beds have more oxygenated conditions and a greater capacity for pathogen elimination [6,7], while deeper beds favor the degradation of wastewater with high organic matter loads due to the predominance of anaerobic conditions [8,9].

Although some studies have examined bed depth, along with factors such as hydraulic retention time (HRT) and aeration rate on pollutant removal in HSSF-CWs [6,8,10,11], there is a lack of information on the performance of these systems when evaluating ornamental species. In addition, most studies with ornamental species have been carried out on a laboratory scale, without fully knowing their performance in real environmental conditions. Moreover, no research has been conducted on the effect that the level of water saturation can have on the development of ornamental vegetation. This is particularly important in tropical areas, where there is a great diversity of ornamental species of commercial interest that could be used in CWs and whose flowers could even be harvested [12]. Therefore, in this study HSSF-CWs with two types of bed depth were planted with ornamental species (*Alpinia purpurata*, *Heliconia latispatha* and *Strelitizia reginae*). The evaluated system was used for the treatment of municipal wastewater; the objective was to determine the influence of said depth on the development of ornamental species and the removal of contaminants. The system was evaluated in real environmental conditions, under tropical climate.

## 2. Methods

### 2.1. Site Description

This study was carried out in the municipality of Misantla, Veracruz, Mexico. The temperature ranged between 13.1 and 27.7 °C. In this region, the mean annual precipitation is between 1900 and 2100 mm; the dry season is from April to June and the rainy season from July to November. The climate of the area is classified as tropical warm-humid, the rainy season is very hot, oppressive and cloudy and the dry season is hot, humid and partially cloudy.

### 2.2. Description of the Experimental System

**Figure 1:**
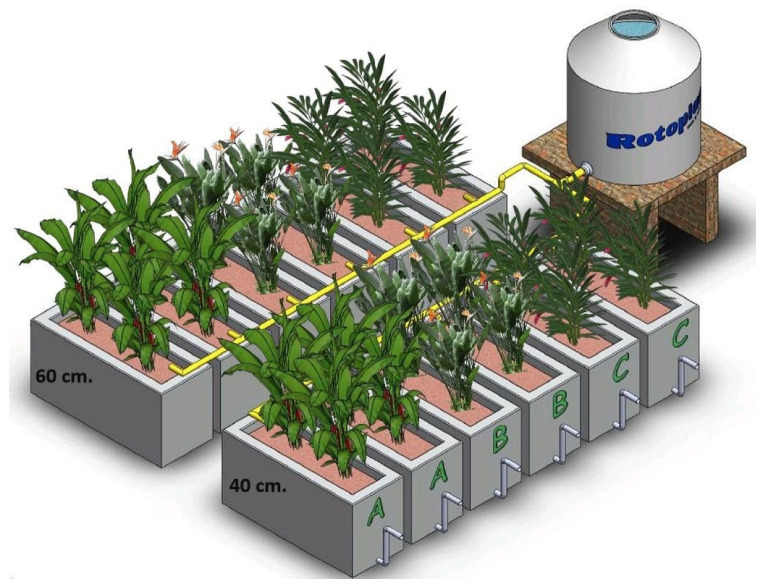
Pilot-scale HSSF-CWs for the evaluation of three ornamental species in monoculture, with two levels of bed saturation. A. *Heliconia latispatha*, B. *Strelitzia reginae* and C. *Alpinia purpurata*.

The wastewater used in the experiment was taken from the final domestic wastewater collector of a neighborhood in Misantla, Veracruz, Mexico. The wastewater was stored in a 1500 L settling tank, which was installed at a height of 0.5 m from the CWs for gravity feeding. The system consisted of 12 cells, constructed of masonry (Figure 1), which were monitored for 24 months. Six units were built with dimensions of 140 cm × 40 cm × 65 cm (length, width and depth), with a water saturation depth of 60 cm. The remaining six units had dimensions of 140 cm × 40 cm × 45 cm, (length, width and depth), with a water saturation depth of 40 cm. Red tezontle of a 5-to-10 mm diameter with a porosity of 0.64 was used as a filter medium. All units had an HRT of 3 days.

**Figure 2:**
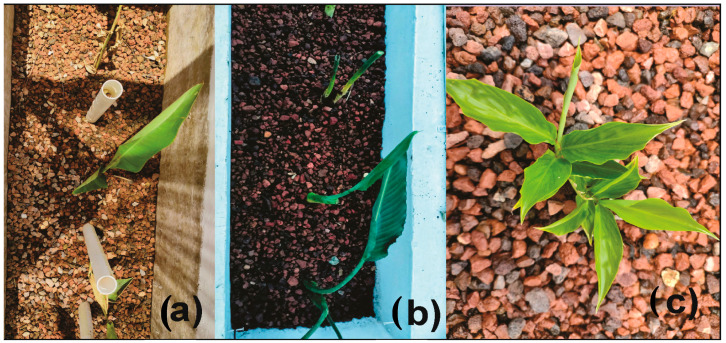
Initial heights of (**a**) *Heliconia latispatha*, (**b**) *Strelitzia reginae* and (**c**) *Alpinia purpurata* at the beginning of the experiment.

Three species of ornamental plants found in their natural state, and which are common in the region, were selected based on previous research that documents their abundance in the area [13,14]. These species were *Heliconia latispatha* (31.6 ± 5.7 cm average height), *Alpinia purpurata* (64.5 ± 3.5 cm average height) and *Strelitzia reginae* (30 ± 1.2 cm average height) (Figure 2). These species were evaluated in monoculture in duplicate cells, with four individuals of each species planted per cell and evenly distributed (Figure 1).

### 2.3. Plant Development

To evaluate vegetation development, the following measurements were taken: plant height and number of leaves (visually counted) every 4 months. Flower counts were conducted monthly to ensure accurate data collection, with a total of 24 measurements over a two-year study period.

Biomass production was measured at the end of the experiment by selecting individuals from each monoculture cell. The procedure and equation used are widely described in Sandoval Herazo [2]. Briefly, individual plants were divided into aboveground and belowground parts, washed with tap water, weighed to obtain fresh weight and then dried to obtain dry weight.

### 2.4. Environmental Parameters and Pollutant Monitoring

Environmental conditions were monitored by taking the ambient temperature and relative humidity measurements three times daily using a digital thermometer. Light intensity was measured between 12:00 and 13:00 h using a Luxmeter model HER-410 (Steren^®^, Mexico City, Mexico). Evapotranspiration was assessed by measuring inlet and outlet flow rates of the cells to calculate pollutant concentrations in effluents. Water temperature, pH and electrical conductivity (EC) were also measured using a portable multiparameter Hanna model HI98121 (HANNA^®^ instruments, Woonsocket, RI, USA), while dissolved oxygen (DO) was measured with a Milwaukee equipment model MW600 (Milwaukee Instruments, Rocky Mount, NC, USA). An adaptation period of 3 months was established before starting the measurement of the selected parameters. After this period, pollutant concentrations were measured in the influent and effluent of each of the cells on a weekly frequency for 24 months, from January 2020 to December 2021. The parameters used to evaluate the quality of the wastewater included COD, TSS, TN, nitrate (N-NO_3_), ammonium (N-NH_4_), total phosphorus (TP), total coliforms (TCs) and fecal coliforms (FCs). The analyses were performed according to standard methods [15].

### 2.5. Experimental Design and Statistical Analysis

Contaminant measurements were carried out weekly, resulting in 104 measurements. Plant development was assessed monthly, with 24 measurements taken over a period of two years. The data obtained were subjected to a two-way analysis of variance (ANOVA) using Statgraphics Centurion XVI. Subsequently, a Tukey mean comparison test was performed with a significance level of 5% (*p* ≤ 0.05). A completely randomized design was employed, where each cell was considered as the experimental unit, with a total of 12 experimental units divided into six treatments varying in saturation levels (60 and 40 cm) and including replicates. In addition, the relationship between nitrogen removal efficiency (N-NO_3_, N-NH_4_ and TN) and the outlet pH and DO values was evaluated using a Pearson correlation analysis.

## 3. Results

### 3.1. Vegetative Development

**Figure 3:**
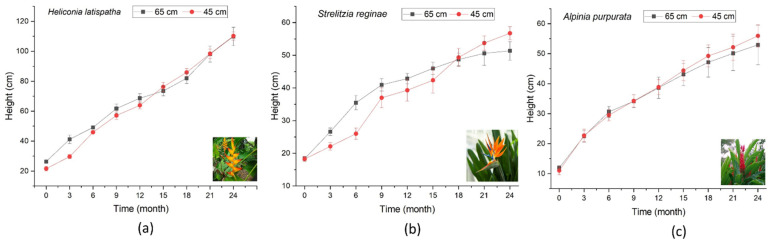
Growth of (**a**) *Heliconia latispatha*, (**b**) *Strelitzia reginae* and (**c**) *Alpinia purpurata* at different bed depths throughout this study.

Figure 3 illustrates the growth of the evaluated plants at two different substrate depths during the 24-month operation period of the CWs.

### 3.2. Heliconia latispatha

*Heliconia latispatha* is native to tropical regions of the Americas and is very common in the study site area. Its extensive root system typically adheres well to different substrates [14]. This species exhibited vigorous growth at both bed depths during the 24 months of this study. The height of the plant showed a sustained increase over time (Figure 3a), and by the third month there was a significant difference (*p* < 0.05) in growth (with a better performance at 0.60 m). At the end of the experiment there was no difference between the heights reached (*p* > 0.05), which were almost 1.2 m. Regarding flower production (Table 1), the number was high with both bed depths, but the number produced was higher with the 0.60 m bed depth (8-to-10 flowers per plant) compared to the 0.40 m bed depth (5-to-7 flowers per plant) (Table 1). Thus, it is confirmed that *Heliconia latispatha* is suitable for use in CWs due to its adaptability with different saturation levels (Figure 3).

### 3.3. Strelitzia reginae

*Strelitzia reginae*, commonly known as the bird of paradise, normally grows to a height of around 90 cm in its natural habitat [16]. However, in this study the maximum height recorded was 0.55 m. From month 1 to 18 the plant presented better development when the saturation depth was 60 cm (*p* < 0.05), while from month 19 to 24 it thrived better at a saturation depth of 40 cm (*p* < 0.05) (Figure 3b). Furthermore, no flowering was observed in the plants (Table 1), probably due to the age of the individuals, since this species usually takes 3-to-5 years to produce flowers.

### 3.4. Alpinia purpurata

**Figure 4:**
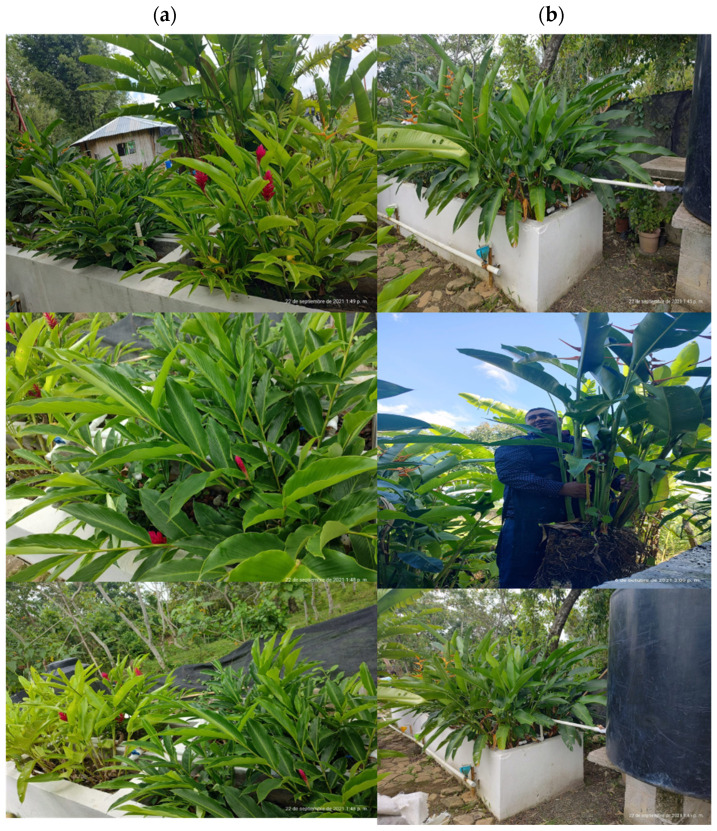
(**a**) *Heliconia latispatha, Strelitzia reginae* and (**b**) *Alpinia purpurata* development.

Throughout this study, *Alpinia purpurata,* also known as Red Ginger, reached a height of up to 0.55 m (Figure 3c) and no significant differences (*p* < 0.05) were found in the development of the species with the two levels of bed depth. Previous studies have shown that *Alpinia purpurata*, despite its extensive coverage that can regulate water temperature and its adaptability to tropical conditions, typically exhibits a low tolerance to water saturation stress (up to a 70 cm depth) and harsh environmental conditions CWs [17]. However, vigorous growth was observed in this study at both depths (Figure 4). Regarding the flowering rate (Table 1), a higher flower production was observed with a bed depth of 40 cm (*p* < 0.05) (Table 2).

In general, the three vegetation species exhibited different behaviors. *Heliconia latispatha* demonstrated prolific flower production, highlighting its adaptability to varying water saturation levels. On the other hand, despite favorable growth, *Strelitzia reginae* did not flower due to its natural characteristics, which do not favor ornamental purposes, as this species typically takes several years to bloom. In comparison, *Alpinia purpurata* reached heights similar to *Strelitzia reginae* but showed greater sensitivity to water saturation stress. The results obtained regarding flower production emphasize that it is a complex process influenced by many factors such as the availability of nutrients (which depend on whether the conditions are aerobic or anaerobic) and the way in which each species or ornamental plant responds. Therefore, the optimal bed depth is different for the flower production for each species of ornamental plant.

### 3.5. Behavior of pH, DO, EC and Temperature in HSSF-CWs

Table 2 shows the average values of pH, DO, EC and temperature at the inlet and outlet points of the evaluated systems. These values are in line with those reported in similar studies using ornamental plants for municipal wastewater treatment [13,18]. A significant reduction in pH (*p* < 0.05) was observed when comparing the inlet and outlet of the CWs cells. However, no significant differences were evident between treatments (*p* > 0.05). The pH can vary due to processes such as nitrification (by producing H+ ions), denitrification (by producing OH–) or anaerobic digestion (which releases organic acids) [19].

On the other hand, a significant increase in DO (*p* < 0.05) was observed in the wastewater after being treated in the systems (Table 2). In the CWs + *Heliconia latispatha* treatment with a depth of 40 cm, a significant difference was found with respect to the other treatments (with an increase of 2.5 mg/L of DO compared to the influent), and in comparison to the CWs + *Heliconia latispatha* treatment with a depth of 60 cm (an increase of 1.7 mg/L with respect to the influent). In general, the results suggest that in shallower systems (40 cm) the conditions tended to be more aerobic. In addition, it was observed that *Heliconia latispatha* developed a greater number of fine roots, which are characterized by releasing more oxygen into the rhizosphere and thus increasing the amount of DO [20,21,22].

Regarding the EC values, the results exhibited a significant decrease (*p* < 0.05) in the effluents compared to the influent, showing a greater reduction in CWs with a depth of 60 cm compared to CWs of 40 cm, regardless of the vegetation type. According to Wang [23], the EC of wastewater treated by CWs may be higher due to a concentration effect resulting from evapotranspiration [24], especially in tropical areas. However, conductivity may also present lower values due to mineral uptake by vegetation.

Regarding temperature, values ranged between 18.2 and 18.6 °C at the 60 cm saturation depth and between 19.1 and 19.4 °C at the 40 cm saturation depth. Notably, in the case of *Alpinia purpurata*, its vigorous growth, extensive coverage and moderate flower production contributed to lower temperatures at the wetland outlet for both saturation depths. These characteristics enable temperature control, preventing water overheating due to prolonged direct sun exposure, which can lead to acidity caused by the rapid degradation of organic compounds at higher temperatures. As expected, temperatures were slightly higher at the shallower 40 cm saturation depth due to an increased exposure to sun and air. This allows solar radiation to penetrate more easily and heat the surface water more effectively [2,10,11].

### 3.6. Pollutant Removal

The efficiency of the system in eliminating the different contaminants is shown in Table 3. The output data in the table comply with the regulations on water quality for discharges to national receiving bodies, as specified by NOM-001-SEMARNAT-2021.

## 4. Discussion

### 4.1. Chemical Oxygen Demand

No significant differences were observed between systems planted with 60 cm and 40 cm saturation levels (*p* > 0.05) for COD removal, nor were there significant differences in COD removal between the species. However, in general, higher COD removal (up to 94%) was achieved in the 60 cm saturation cells. This difference is mainly attributed to the presence of anoxic/anaerobic conditions in deeper cells, as evidenced by lower DO values at the outlet. The performance of these systems coincides with findings from previous studies, such as the one carried out by Pascual et al. [6]. The authors evaluated different depths of bed saturation and found COD removals greater than 90% in deeper CWs. Similarly, the study carried out by Ren et al. [10] found a higher COD removal in systems with a substrate depth of 0.6 m.

### 4.2. TSS

The results obtained showed that this horizontal flow system provided favorable conditions for the degradation and filtration of suspended particles. For both 40 cm and 60 cm saturation depths, removals were above 80%, indicating effective filtration due to the substrate and the plants’ adaptability. At 60 cm depth, *Heliconia latispatha* exhibited a slightly higher TSS removal compared to the other two species, with removals of 81.2% and 70.39% at 40 cm depth. Due to its vigorous growth and an extensive root system that adheres well to different substrates and promotes biofilm formation, this species demonstrated a high capacity to retain and filter suspended solids in water. The results obtained were higher than those reported by Sandoval et al. [2] in a study with monoculture and polyculture where *Heliconia latispatha* had an average removal of 54.9% at a depth of 65 cm. Similarly, this plant is characterized by its high adaptability to different substrates such as PET, where it was evaluated in a study by Fernández-Echeverría et al. [14], obtaining removals of 55.5% of TSS. On the other hand, *Strelitzia reginae* had a removal of 72.5% at a depth of 40 cm, slightly higher than the other species. Despite not flowering during this study, it exhibited vigorous development, which also contributes to TSS retention. In the case of *Alpinia purpurata*, despite its sensitivity to water saturation stress, it also showed good performance in removing TSS, reaching values of 77.5% for 60 cm and 71.8% for 40 cm. This can be attributed to its dense coverage and adaptability to tropical conditions [17]. The variability in removal efficiencies between different depths can be attributed to nutrient availability and water oxygenation, which also affect the plant’s ability to thrive and maintain water quality in CWs [22].

### 4.3. Nitrate

N-NO_3_ removal is mainly carried out through denitrification, which requires anoxic/anaerobic conditions; such conditions are determined by the bed saturation level and the type of vegetation species, as found in this research. A significant difference was found among the species evaluated (*p* < 0.05), and the systems planted with *Strelitzia reginae* recorded the best performance in N-NO_3_ removal, with no impact of bed saturation level (*p* < 0.05). In contrast, in systems planted with *Heliconia latispatha* and *Alpinia purpurata* with a saturation level of 40 cm, significantly low values in N-NO_3_ removal were observed compared to deeper systems. This behavior can be explained, as it is likely that aerobic zones facilitated nitrification, converting N-NO_2_ to N-NO_3_, but conditions were not suitable for denitrification, resulting in nitrate accumulation [25,26,27].

### 4.4. Ammonium

The type of vegetation and the level of saturation of the bed significantly influenced the ammonium reduction. This was observed for all ornamental species evaluated. For example, *Heliconia latispatha* systems achieved higher removal rates (90.3% ± 0.2) with 40 cm saturation compared to 60 cm saturation (69.7% ± 0.4) (*p* < 0.05). In a shallower substrate (40 cm), the aerobic conditions were favorable for the activity of nitrifying bacteria, as seen in Table 1. Deeper beds, with common anoxic conditions, limited the autotrophic nitrifying bacteria presence, thereby reducing ammonium removal through nitrification [19]. Other studies have already reported the influence of ornamental species on N-NH_4_ removal, which can be attributed to the different uptake capacities and demands of each species. For example, Sandoval et al. [25] reported a 46.5% removal of N-NH_4_ with *S. blandum* at the mesocosm scale and 89.3% with *M. aquaticum*.

### 4.5. Total Nitrogen

TN removal in CWs primarily occurs via plant uptake, mineralization, sedimentation, nitrification–denitrification and microbial uptake mechanisms [28]. In this study, the removals varied from 71.9% to 86.3% without a significant difference between plant species and bed depth (*p* > 0.05). However, overall, TN removals were lower in systems with 60 cm saturation compared to those with 40 cm saturation. Although not clearly significant, the highest removals were observed in the *Strelitzia reginae* system (in both bed depths), achieving values of 79.4% and 86.3% for 60 cm and 40 cm depths, respectively. Greater nitrate removal in *Strelitzia reginae* systems may explain the higher overall removals. TN removal in HSSF-CWs can be limited in deeper substrate systems due to reduced root–microorganism interactions, resulting in lower nitrification rates. The limited available oxygen is mainly used by heterotrophic bacteria for the degradation of organic matter. Furthermore, the metabolic activity of aerobic heterotrophs can deplete the carbon sources needed by denitrifying bacteria for nitrate reduction and subsequent TN reduction [29].

In general, the results of nitrogen component removal (TN, N-NH_4_ and N-NO_3_) and the pH and DO values demonstrated a relationship between these parameters. The correlation between ammonium removal and effluent pH is positive (0.786), suggesting that a slightly higher pH (while remaining neutral) could enhance ammonium removal. For example, in the case of *Alpinia purpurata* a higher ammonium removal (85.1%) is observed at a depth of 40 cm and a pH of 7.2, compared to 60.9% removal at a depth of 60 cm and a pH of 6.8. This suggests that a greater saturation depth slightly reduced the pH, resulting in lower ammonium removal in this plant [10,13].

Similarly, for TN the correlation is positive (0.798) and moderately strong, supporting the notion that a slightly higher pH may improve overall nitrogen removal. Regarding DO, the correlation between nitrate removal and DO is negative (−0.252), but weak and not significant, indicating no clear relationship between DO and nitrate removal efficiency. This is likely because the variability in DO does not affect denitrifying bacteria, which are anaerobic. On the other hand, the correlation between ammonium removal and DO output is positive (0.605) and moderate, suggesting that higher dissolved oxygen levels are associated with greater ammonium removal. This is evident in the case of *Heliconia latispatha* at a depth of 40 cm, where a higher ammonium removal (90.3%) is observed with a DO of 4.3 mg/L, compared to 69.7% removal at 60 cm depth with a DO of 3.5 mg/L [19,22].

### 4.6. Total Phosphorus

TP removals did not exceed 75%; however, this is a fairly high removal efficiency, considering that TP removal in CWs is usually low. The main ways of phosphorus removal in CWs are adsorption processes with the filter medium, chemical precipitation and adsorption by microorganisms and plants. The DO content influences TP removal, as an aerobic environment enhances the precipitation and adsorption of phosphorus on the substrate [30]. Additionally, the phosphorus removal efficiency increases with a decreasing size of the support material because it increases the specific surface area of the material and, consequently, the adsorption sites [31,32]. The plant that showed the best performance in TP removal was *Heliconia latispatha* with the two saturation levels (*p* < 0.05). On the contrary, CWs with *Strelitzia reginae* presented the lowest performance. It is probable that the difference between the cells planted with these two species was due to the vigorous growth of *Heliconia lathispatha* (with the production of several flowers), which implied a higher phosphorus uptake, confirming that under tropical conditions direct uptake by plants plays an important role as a removal mechanism, which has also been reported by Sandoval et al. [2].

### 4.7. Total Coliforms and Fecal Coliforms

*Heliconia latispatha* cells at both depth levels exhibited the highest bacteriological load reduction efficiencies compared to other plants (Table 3); however, they achieved removals of only 57.9% ± 0.3% (0.40 cm) and 60.6% ± 0.3% (0.60 cm). Cells planted with *Strelitzia reginae* showed the lowest removal efficiencies, with significant differences (*p* < 0.05) observed for both TCs and FCs. Overall, removal results were slightly higher in the shallower systems (40 cm), likely due to an increased dissolved oxygen availability in the rhizosphere released by the plants. It is documented that aerobic conditions are conducive to the removal of pathogens measured as TCs and FCs [33]. Consistent with this, Headly et al. [34] demonstrated significantly higher removal rates of TCs in CWs with aerated surfaces compared to non-aerated surfaces, evidencing the positive impact of DO on FCs removal efficiency. Another important factor in the fecal microorganism removal during the treatment of CWs in tropical regions is the influence of temperature, solar radiation, sedimentation and filtration [35].

### 4.8. Plant Development

The growth of *Heliconia latispatha* (Figure 2) and flower production (Table 1) was lower in this study than in the study by Fernandez et al. [14], where *Heliconia latispatha* plants reached a height of up to 217.5 ± 10 cm with a saturation level of 0.65 m, using PET and tezontle as filter medium; however, this was likely due to the higher concentration of phosphorus and N-NH_4_ in the swine wastewater that the authors evaluated.

*Strelitzia reginae* did not present floral development at any of the depths (Table 1), while in a study by Zurita et al. [12] three of six *S. reginae* plants flowered in HSSF-CWs with a saturation depth of 30 cm, while five of six plants produced larger flowers in vertical CWs. Another study by Lara-Acosta et al. [36] demonstrated that *S. reginae* can adapt to wastewater conditions, although no floral growth was observed.

Finally, in this study *Alpinia purpurata* produced flowers (Table 1). This could be because the red volcanic gravel filter medium presents additional nutrients and minerals that can favor floral development, while in a study by Sandoval-Herazo et al. [37], in which PET substrates and depths ranging between 50 and 60 cm were evaluated, *Alpinia purpurata* did not produce flowers, probably due to the short evaluation period.

## 5. Conclusions

According to the findings of this study, the saturation level in the CWs (40 cm or 60 cm) can influence the development of ornamental vegetation, particularly flower production. *Heliconia latispatha* exhibited higher flowering rates at 60 cm saturation, while *Apinia purpurata* showed higher flowering rates at 40 cm saturation. This highlights the importance of selecting the appropriate saturation level based on the ornamental species. Regarding contaminant removal, the impact of the saturation level varies depending on the specific contaminant. In general, the depth of 60 cm saturation favored the removal of COD, with removal efficiencies of 94% for the three plant species. The depth of 40 cm was the most effective for the removal of N-NH_4_ (80–90%). Regarding the removal of TN, the removals were similar for the different plants and depths (72–86%).

The use of *Heliconia latispatha*, *Strelitzia reginae* and *Alpinia purpurata* is suggested as vegetation in CWs, although *Strelitzia reginae* did not manage to flower, and so its evaluation is recommended over longer periods of time to establish flowering conditions and health of the same level on CWs. Studies with polycultures of these tropical plants would be desirable to establish if together they increase the elimination of some specific contaminants and the best combinations between them to improve the performance of the CWs. It is recommended that the 40 cm depth in combination with any of the three plants be used to treat municipal wastewater that can later be used for the irrigation of gardens or agricultural crops.

In general, CWs efficiency is usually satisfactory in tropical regions due to favorable temperatures. Ornamental plants contribute significantly to the removal of pollutants, particularly nitrogen and phosphorus.

## Figures and Tables

**Table 1:** Total flower production in each system.

Month	60 cm			40 cm		
	*Heliconia* *latispatha*	*Alpinia* *purpurata*	*Strelitzia* *reginae*	*Heliconia* *latispatha*	*Alpinia purpurata*	*Strelitzia* *reginae*
12	65	54	0	48	92	0
24	75	41	0	51	65	0
Total	140	95	0	99	157	0

**Table 2:** Average pH, DO, EC and temperature values in HSSF-CWs with varied depths and vegetation.

Parameter	60 cm						40 cm					
	*Heliconia latispatha*		*Alpinia purpurata*		*Strelitzia reginae*		*Heliconia latispatha*		*Alpinia purpurata*		*Strelitzia reginae*	
pH												
Input	7.8 ± 0.2											
Output	6.8 ± 0.1		6.8 ± 0.2		6.9 ± 0.1		7.1 ± 0.1		7.2 ± 0.1		7.1 ± 0.2	
DO (mg/L)												
Input	1.8 ± 0.3											
Output	3.5 ± 0.2		2.2 ± 0.3		3.1 ± 0.2		4.3 ± 0.2		2.9 ± 0.3		3.9 ± 0.3	
EC (µs/cm)												
Input	1002.6 ± 82.9											
Output	1034.7 ± 94.4		1103.2 ± 78.7		1192.7 ± 106.5		1206.6 ± 67.8		1319.08 ± 99.2		1283.4 ± 58.2	
Temperature (°C)												
Input					25.4 ± 3.7							
Output	18.4 ± 0.5		18.2 ± 0.9		18.6 ± 0.3		19.3 ± 0.5		19.1 ± 0.2		19.4 ± 0.2	

**Table 3:** Efficiency of HSSF-CWs with three plant species and two bed depths for the removal of different pollutants.

Saturation	60 cm						40 cm					
Parameter	*Heliconia latispatha*		*Alpinia purpurata*		*Strelitzia reginae*		*Heliconia latispatha*		*Alpinia purpurata*		*Strelitzia reginae*	
COD (mg/L)												
Influent	175.3 ± 1.4											
Effluent	10.5 ± 8.1		10.6 ± 4.1		10.5 ± 2.2		32.0 ± 7.9		24.7 ± 1.6		26.7 ± 3.6	
Removal (%)	94.0 ± 7.4		93.9 ± 8.1		94.0 ± 8.4		81.7 ± 4.6		85.9 ± 17.9		84.8 ± 24.4	
TSS (mg/L)												
Influent					110.83 ± 4.62							
Effluent	20.84 ± 5.3		24.94 ± 6.3		24.38 ± 4.5		32.82 ± 4.8		31.25 ± 9.7		30.48 ± 4.1	
Removal (%)	81.2 ± 4.7		77.5 ± 6.3		78 ± 7.1		70.39 ± 4.2		71.8 ± 8.3		72.5 ± 9.2	
N-NO_3_ (mg/L)												
Influent	14.1 ± 0.2											
Effluent	4.3 ± 0.1		2.9 ± 0.1		1.0 ± 0.1		6.1 ± 0.2		4.7 ± 0.2		1.0 ± 0.1	
Removal (%)	69.2 ± 0.7		78.7% ± 0.6		92.6 ± 0.3		57.3 ± 1.3		66.3 ± 1.2		92.3 ± 0.3	
N-NH_4_ (mg/L)												
Influent	9.33 ± 0.066											
Effluent	2.8 ± 0.1		3.6 ± 0.1		4.4 ± 0.2		0.9 ± 0.1		1.4 ± 0.1		1.9 ± 0.2	
Removal (%)	69.7 ± 0.4		60.9 ± 0.4		53.3 ± 0.3		90.3 ± 0.2		85.1 ± 0.2		80.1 ± 0.2	
TN (mg/L)												
Influent	56.5 ± 0.1											
Effluent	15.9 ± 0.8		13.4 ± 0.3		11.6 ± 0.4		10.8 ± 0.5		10.5 ± 0.5		7.8 ± 0.3	
Removal (%)	71.9 ± 1.4		76.3 ± 0.5		79.4 ± 0.7		81.0 ± 0.9		81.4 ± 0.8		86.3 ± 0.6	
TP (mg/L)												
Influent	10.3 ± 0.2											
Effluent	3.6 ± 0.1		4.5 ± 0.2		5.5 ± 0.1		2.7 ± 0.1		3.4 ± 0.1		4.6 ± 0.2	
Removal (%)	64.7 ± 1.2		55.6 ± 1.5		46.5 ± 1.4		73.1 ± 1.1		65.9 ± 0.9		55.4 ± 1.2	
TCs (MPN/100) mL												
Influent	6394.3 ± 62.5											
Effluent	2686.6 ± 8.2		2698.7 ± 7.8		3404.9 ± 16.3		2515.6 ± 8.7		2816.4 ± 9.4		3323.6 ± 26.7	
Removal (%)	57.9 ± 0.3		57.7 ± 0.4		46.6 ± 0.5		60.6 ± 0.3		55.8 ± 0.4		47.9 ± 0.5	
FCs (MPN/100) mL												
Influent	3010.73 ± 21.1											
Effluent	1340.1 ± 4.5		1348.7 ± 4.6		1808.6 ± 14.4		1265.1 ± 4.2		1410.8 ± 4.3		1649.2 ± 11.1	
Removal (%)	55.4 ± 0.3		55.2 ± 0.4		39.9 ± 0.5		57.9 ± 0.2		53.1% ± 0.4		45.2 ± 0.4	

## Data Availability

All relevant data are included in this paper. Additional data supporting the conclusions can be requested ffrom the corresponding author.
